# Supplementary material for: Definitions of successful aging among middle-aged Latinas residing in a rural agricultural community
Source: PLoS One. 2023 Nov 30;18(11):e0294887. doi: 10.1371/journal.pone.0294887 (PMC10688629; doi:10.1371/journal.pone.0294887)
Supplement: S1 Appendix — (DOCX) [file pone.0294887.s002.docx]

**Appendix A. Focus Group Guide**

**Aim 1. To examine definitions of “successful aging.”**

1. What comes to mind when you hear the words “successful aging”?
   1. What does “successful aging” mean to you?
2. Can you describe someone you know who you think is “aging successfully”?
   1. Why do you think they are “aging successfully”, as opposed to someone who is not?
3. Do you think you are “aging successfully”? Why? If not, why not?
   1. Are there any healthy habits that you’ve used throughout your life?
   2. What helps you or would help you stay well?
4. When you think about the people you mentioned and yourself, what do you think a person needs to do to “age successfully”?
5. What barriers do you experience to “age successfully”?
   1. What makes it difficult for you to “age successfully”?
6. What would make you satisfied in old age?
   1. What would be your ideal situation when you are 65 and older?
   2. What resources do you think you will need to experience a good quality of life when you are older?
   3. Do you think you’ll be able to get the resources you need?
7. What worries you about “aging”?
   1. What things do you need to do now to prepare for “successful aging”?
