# Supplementary material for: Definitions of successful aging among middle-aged Latinas residing in a rural agricultural community
Source: PLoS One. 2023 Nov 30;18(11):e0294887. doi: 10.1371/journal.pone.0294887 (PMC10688629; doi:10.1371/journal.pone.0294887)
Supplement: Attachment — Submitted filename: Report on Ambriz et al_Resubm.pdf [file pone.0294887.s004.pdf]

Ambritz and colleagues present a qualitative study on 'successful aging' from the perspective of Latina women residing in a rural community. The group under study demographically corresponds to 40 Latina women from a rural agricultural community, ethnically predominant Mexican, within a middle age range of 40-49 (60%) and 50-59 (35%), 70% of them employed and 30% unemployed. The goal of the study was to investigate the perspectives on 'successful aging' by this group of Spanish speaking women, methodologically conducted by a facilitator in focus groups, organized by age, using as a guide a questionnaire of 7 major questions about 'successful aging', and contextual variations around each one of them, aiming at developing a more complete picture of the viewpoints and perceptions on the topic by the group under investigation. The conversations, lasting 2hs in each focus group were recorded and later transcribed.
Good health and acceptance of body changes associated with aging, active lifestyle, children wellbeing and independence were the major components of successful aging, as perceived by the women group investigated.

Major comment
The premise of the study of highlighting the role played by psycho-social-cultural factors involved in the perceptions of 'successful aging', and its findings as potential tools to promote and guide policies leading to achieve healthy aging, are valuable complements of the biological perspective on aging.
Although the present study raises an important point, for instance, the impact on the quality of life of the participating women exerted by their children wellbeing, it is also true that if something aggravating happens to a child it will be followed by an impact on mothers' biology, e.g., immune system, making them more prone to disease and degradation of life quality. The lack of explicit recognition of the tight interconnections between individual/group perceptions of successful aging and the underlying biology of health and disease is what this paper is missing. This recognition strengthen rather than weakening the premise of the study while recognizing the complexity of the topic, i.e., 'successful aging', that this paper approaches. This is why this Reviewer notes that the approach utilized in this study is a 'valuable complement' of the biological perspective on aging.
In regard of this, a recent investigation on cognitive decline and dementia in the Multi-Ethnic Study of Atherosclerosis (MESA) comprising 1036 participants of Black, Chinese-American, Hispanic and White origin, of which 53% were women, showed that it is not race/ethnicity but cardiovascular risk factors such as age, smoking, hypertension, diabetes and socio-economic status, that determine the outcome on cognitive decline (Austin, T.R. et al., 2022. JAHA).
