# Supplementary material for: Definitions of successful aging among middle-aged Latinas residing in a rural agricultural community
Source: PLoS One. 2023 Nov 30;18(11):e0294887. doi: 10.1371/journal.pone.0294887 (PMC10688629; doi:10.1371/journal.pone.0294887)
Supplement: Attachment — Submitted filename: Response to Reviewers.docx [file pone.0294887.s003.docx]

February 23, 2023

Dear Dr. Chennet,

Thank you for your invitation to revise and resubmit our manuscript, “Definitions of successful aging among middle-aged Latinas residing in a rural agricultural community” for *PLOS ONE.*

We greatly appreciate the time and thoughtful comments from the three reviewers and have revised our manuscript accordingly. We have provided a point-by-point response to each critique below. We believe that our manuscript has been strengthened substantially as the result of these suggested revisions.

Sincerely,

The authors

**Response to Reviewers**

Journal Requirements:

When submitting your revision, we need you to address these additional requirements.

1. Please ensure that your manuscript meets PLOS ONE's style requirements, including those for file naming. The PLOS ONE style templates can be found at

[https://journals.plos.org/plosone/s/file?id=wjVg/PLOSOne_formatting_sample_main_body.pdf](https://urldefense.com/v3/__https:/journals.plos.org/plosone/s/file?id=wjVg*PLOSOne_formatting_sample_main_body.pdf__;Lw!!LQC6Cpwp!r-DdYexZeh0hoZSpJXRyvJ_RcmWTUOp4_tgP2Fbrg553vFS-vjyVEfZKkONi32QiiGTFobK23WYoDtPyI6n6ejb5$)  and

[https://journals.plos.org/plosone/s/file?id=ba62/PLOSOne_formatting_sample_title_authors_affiliations.pdf](https://urldefense.com/v3/__https:/journals.plos.org/plosone/s/file?id=ba62*PLOSOne_formatting_sample_title_authors_affiliations.pdf__;Lw!!LQC6Cpwp!r-DdYexZeh0hoZSpJXRyvJ_RcmWTUOp4_tgP2Fbrg553vFS-vjyVEfZKkONi32QiiGTFobK23WYoDtPyIz2lccJ4$)

**Response:** We have correctly formatted the manuscript and the title page.

2. We note you have included a table to which you do not refer in the text of your manuscript. Please ensure that you refer to Table 1 in your text; if accepted, production will need this reference to link the reader to the Table

**Response:** We have included reference to table 1 in the text.

3. Thank you for stating the following financial disclosure:

“All authors are supported by the National Institute on Aging R01069090.”

Please state what role the funders took in the study.  If the funders had no role, please state: "The funders had no role in study design, data collection and analysis, decision to publish, or preparation of the manuscript." If this statement is not correct you must amend it as needed. Please include this amended Role of Funder statement in your cover letter; we will change the online submission form on your behalf.

**Response:** We have added the financial disclosure statement stating that funders had no role.

4. In your Data Availability statement, you have not specified where the minimal data set underlying the results described in your manuscript can be found. PLOS defines a study's minimal data set as the underlying data used to reach the conclusions drawn in the manuscript and any additional data required to replicate the reported study findings in their entirety. All PLOS journals require that the minimal data set be made fully available. For more information about our data policy, please see [http://journals.plos.org/plosone/s/data-availability](https://urldefense.com/v3/__http:/journals.plos.org/plosone/s/data-availability__;!!LQC6Cpwp!r-DdYexZeh0hoZSpJXRyvJ_RcmWTUOp4_tgP2Fbrg553vFS-vjyVEfZKkONi32QiiGTFobK23WYoDtPyIw4usDs3$).

Upon re-submitting your revised manuscript, please upload your study’s minimal underlying data set as either Supporting Information files or to a stable, public repository and include the relevant URLs, DOIs, or accession numbers within your revised cover letter. For a list of acceptable repositories, please see [http://journals.plos.org/plosone/s/data-availability#loc-recommended-repositories](https://urldefense.com/v3/__http:/journals.plos.org/plosone/s/data-availability*loc-recommended-repositories__;Iw!!LQC6Cpwp!r-DdYexZeh0hoZSpJXRyvJ_RcmWTUOp4_tgP2Fbrg553vFS-vjyVEfZKkONi32QiiGTFobK23WYoDtPyI3_0kfoy$). Any potentially identifying patient information must be fully anonymized.

Important: If there are ethical or legal restrictions to sharing your data publicly, please explain these restrictions in detail. Please see our guidelines for more information on what we consider unacceptable restrictions to publicly sharing data: [http://journals.plos.org/plosone/s/data-availability#loc-unacceptable-data-access-restrictions](https://urldefense.com/v3/__http:/journals.plos.org/plosone/s/data-availability*loc-unacceptable-data-access-restrictions__;Iw!!LQC6Cpwp!r-DdYexZeh0hoZSpJXRyvJ_RcmWTUOp4_tgP2Fbrg553vFS-vjyVEfZKkONi32QiiGTFobK23WYoDtPyI6h-UsvO$). Note that it is not acceptable for the authors to be the sole named individuals responsible for ensuring data access.

We will update your Data Availability statement to reflect the information you provide in your cover letter.

**Response:** The focus group data hold potentially identifying sensitive information regarding the participants, and it would therefore be unethical to make this public and would undermine the minimal risk ethical committee agreement and consent process. Please contact the University of California, San Francisco Institutional Review Board (IRB) to request anonymized focus group data access at [CHR@ucsf.edu](mailto:CHR@ucsf.edu).

5. Please include captions for your Supporting Information files at the end of your manuscript, and update any in-text citations to match accordingly. Please see our Supporting Information guidelines for more information: [http://journals.plos.org/plosone/s/supporting-information](https://urldefense.com/v3/__http:/journals.plos.org/plosone/s/supporting-information__;!!LQC6Cpwp!r-DdYexZeh0hoZSpJXRyvJ_RcmWTUOp4_tgP2Fbrg553vFS-vjyVEfZKkONi32QiiGTFobK23WYoDtPyI_VLk1h-$).

**Response:** We included citations for our supporting information files and updated in-text citations to match accordingly.

6. Please ensure that the study is reported according to the COREQ guideline, and include the completed COREQ checklist as Supporting Information. Please add the following statement, or similar, to the Methods: "This study is reported as per the Consolidated criteria for reporting qualitative research (COREQ) guideline (S1 Checklist)."

The COREQ guideline can be found here: [https://www.equator-network.org/reporting-guidelines/coreq/](https://urldefense.com/v3/__https:/www.equator-network.org/reporting-guidelines/coreq/__;!!LQC6Cpwp!r-DdYexZeh0hoZSpJXRyvJ_RcmWTUOp4_tgP2Fbrg553vFS-vjyVEfZKkONi32QiiGTFobK23WYoDtPyI1c-KC2R$) 
When completing the checklist, please use section and paragraph numbers, rather than page numbers.

**Response:** We added the statement about COREQ to the methods and included the completed COREQ checklist as supporting information.

Additional Editor Comments:

The Data Availability Statement (DAS) requires revision. If the data are freely or publicly available, please include the DOI or accession number).

**Responses to Reviewers' comments:**

**Reviewer #1**: I enjoyed reading this well-written manuscript, and found the results to be interesting and insightful. The study methods sound appropriate, and the findings provide new insights into Latino perspectives on successful ageing. I would very much like to see this work published, although I do think that there are a few issues that the authors should consider before the manuscript is ready for publication. I outline these below.

Main points

1. Insufficient attention is currently paid to the fact that the study only includes female participants. At the very least, this should be explained and justified in the introduction section. It is also something that could be worth exploring in the discussion section – especially when considering how the current findings differ to those of previous work in Latino populations e.g. what proportion of previous samples were female, and could the smaller proportions of female participants (or, indeed, the method of data collection e.g. if mixed gender focus groups were used) account for the differences in key themes that emerged? Based on these reflections, do you feel that gender differences should be explored further in similar research?

**Response:** We updated our introduction to explain why studying Latina females is needed. We also added a comment in the discussion section and suggested that gender differences should be explored further in similar research.

2. Your description of the analytical procedure is a little unclear, and lacks important detail. For instance:
a. What kind of coding did you use (and might you consider adding a table with some examples of the initial codes that you applied to some of the quotes from each theme?)?
b. What do you mean by ‘narratives’ and how do these differ from your themes?
c. More detail is needed to explain how you did your collaborative coding, and how you used the Consolidated Framework.

**Response:** We added important detail about the coding process to the methods section.

3. There is a fair bit of overlap between your first two themes, in that Theme 1 (‘Having Good Health and Accepting Changes in the Physical Body’) includes details of participants wanting to maintain active lifestyles, and to continue to take part in valued activities, and Theme 2 (‘Maintaining an Active Lifestyle’) then ends by mentioning participants’ desires to maintain their health and wellbeing. Whilst these two themes are logically related to one another, I felt that the content and narratives could be better organised so that there is a clearer distinction between the focus of each.

**Response:** We reorganized the content and quotes to make a clearer distinction between theme 1 and theme 2.

4. Whilst the introduction and discussion do a great job of considering what is known about Latino peoples’ perspectives on successful ageing, I think that the paper could be much improved by also considering how these findings compare to findings from other cultures and groups. For instance, which of the findings seem to be somewhat universal across cultures/groups, and which are more specific to Latinos, or to other groups with shared characteristics? For instance, work in this area conducted with Chinese immigrants to a Western country by Teh et al (2020: DOI: 10.1111/ajag.12677) found that participants saw their offspring’s success and wellbeing as integral to their own sense of ageing well. The work also highlights a similar tension between participants wanting their children to be independent, whilst also feeling concern at them not being there to take care of them as they age. It would therefore be useful to situate the current findings within this broader literature base so that such similarities and differences are highlighted.

**Response:** We cited the findings from Teh et al and Nguyen H et al comparing definitions of successful aging among Korean, Vietnamese, and Latino older adults in the discussion section to situate the current findings within this broader literature.

5. In relation to the previous point, your argument in the discussion about the ‘unique contribution’ of ‘the important role of children’ that this work makes to ‘successful ageing frameworks’ is not accurate, as these points have already been made by authors such as Teh and colleagues.

**Response:** We reframed this argument to say that we’re reinforcing previous findings about ‘the important role of children.’

Minor points

6. There are a couple of missing bits of text in the version of the manuscript that I saw. In particular:
a. The introduction paragraph of the abstract contains a question mark in place of a word (‘..rural Latinos experience more structural barriers than ? to access the conditions…’)
b. The penultimate sentence of the results section ends with empty quotation marks (‘This was illustrated by one participant as ‘’ ‘).

**Response:** We corrected these typos.

7. It would be useful to state the overall age range of the participants, rather than just the mean age, in the abstract, introduction, and methods section.

**Response:** We added the overall age range of participants to the abstract, introduction, and methods section.

8. The first sentence of the second paragraph of the Background section (starting: ‘Latinos, especially rural Latinos, in the United States…’) should be supported with a citation.

**Response:** We added a citation to this section.

9. It would be useful to know in the methods section how and why the particular subset of 70 responders were chosen from the 202 that contacted the research team.

**Response:** We added more detail about reasoning for following up with the 70 responders.

10. The final couple of sentences of text in the ‘participants section’ (that describe the size of the focus groups, the dates of data collection, and the means of ethical approval) would seem better placed in the ‘data collection’ section.

**Response:** We moved the sentences describing data collection to the data collection section.

11. I would suggest providing a more complete description of the topic guide in the main manuscript, as many readers will download the paper without the appendix, and so will not have access to this when reading the paper.

**Response:** We added a more detailed description of the semi-structured interview guide to the manuscript.

12. Could you explain why you chose to organize focus groups by age?

**Response:** We explained the reasoning behind organizing the focus groups by age, which is to explore/understand age differences in definitions of successful aging. We added these age differences to the findings.

13. Some of the information in the demographics table is not useful. In particular, as you explain elsewhere that all of the participants were female, and that they all spoke Spanish, it doesn’t seem necessary to report the %s of these characteristics. I also felt that these demographic details of your sample might be better placed in your participants section, than your results, as they are descriptions of your sample rather than ‘findings’.

**Response:** We updated our demographic table to remove redundant data and moved it to the participants section.

14. In the discussion section you mention that all of the participants had children. If this information was formally collected as part of the study, then it would be useful to report this when describing the characteristics of the sample.

**Response:** We report this when describing the CHAMACOS cohort, from which we drew our sample.

15. It is not clear what you mean in the ‘strengths and limitations section by your sample being ‘robust’.

**Response:** We reframed the strengths and limitations sections to highlight the unique composition of our sample including more than half agricultural workers.

16. If word count is an issue, then I think that much of the first paragraph of the discussion could be omitted, as it repeats many points from the introduction. Other parts of the discussion also reiterate parts of the results section that are not needed. Some of your quotes could also be cut down.

**Response:** We significantly edited the first paragraph of the discussion to remove redundant content.

**Reviewer #2:** This is a well-written paper on successful aging in Latinas in a rural community. This paper contributes to the literature by providing the perspectives of a unique population. I have some minor comments below for the authors to consider in revising their manuscript.

Introduction: The authors build a strong case for investigating successful aging in middle-aged Latinos. However, the introduction is missing some context as it pertains to women specifically, and related to the life expectancy of Latinas. These pieces are need to provide a rationale for the sample, and to provide context to the results.

Line 160: How did the researchers decide which participants to follow-up with? Why didn’t they follow-up with all participants? Some explanation is required here to provide more understanding of bias in the sample selection.

**Response:** We added more detail about reasoning for following up with the 70 responders. We conducted focus groups until saturation was reached and referred all the interested responders to another ongoing qualitative study.

Line 163: How was it established that saturation was reached?

**Response:** We explained that saturation was reached when no new themes emerged from the data.

Table 1: Since only women were recruited, gender can be removed from the table. Also, did the authors ask about gender, or are they using biological sex?

**Response:** We updated the demographics table and removed gender and language. We are using biological sex.

Results: Since the focus groups were done in age-groups, can the authors address age differences in the findings? This comparison would help elucidate some of the issues or gaps that were identified in the introduction of the paper.

**Response:** We highlighted the key age differences in the findings, particularly in theme 1.

Results: Quotes should be pulled out from the paragraphs to improve readability and to highlight them.

**Response:** We tried to pull the quotes from the paragraphs to improve readability, but they looked choppy, and we didn’t think it improved readability. However, we defer to the editor on this.

Results: The high-level themes appear to have several sub-themes that are not clearly addressed. For example, it appears that weight came up on more than on occasion. Is there a sub-theme related to weight that might need to be highlighted? Similarly, some of the quotes suggest that perhaps the women had low self-esteem or were depressed. But perhaps there is a cultural component pertaining to humility that is being captured in these quotes instead. It would be nice to see a more thorough breakdown of the data. A table or figure could clearly provide the themes and sub-themes. This would also help with highlighting the novelty in the discussion as well i.e. cultural differences, age differences, etc.

**Response:** We highlighted several sub-themes under the high-level themes. We added a table to the results section summarizing the themes and subthemes with exemplar quotes.

Introduction and Discussion: Given that the age of the sample was much younger than samples who are typically included in studies regarding successful aging, it is important for the authors to highlight the sample of the age in the studies being used for comparison. Are there other studies that have asked about successful aging in middle-aged adults that can be used for direct comparison? It is also worth addressing the fact that the probing questions used across studies can vary significantly. For example, the current study included a question asking for examples of people who were aging successfully. Other studies may not have asked this directly, and therefore it may not have arisen as a theme. Comparison to previous literature needs to be put in the context of the age of the sample and the probing questions used.

**Response:** We highlight the sample of the age in the studies being used for comparison in the introduction, those 60 and over. We did not find other similar studies that have asked about successful aging in middle-aged adults that can be used for direct comparison.

Line 366: Did all of the women in the sample have children? If such a dimension is added to a framework, will it be inclusive of such women? I’m not sure I agree with the suggestion being made in this line.

**Response:** Yes, we described in the participant description that all the women in the sample have children. We edited 366 and instead highlighted that this finding reinforces previous findings including Chinese immigrants about the role of children on parental wellbeing and their own definitions of successful aging.

Discussion: The rural aspect of the sample did not appear to come through in the discussion, other than in the section on physical demands of work. This is a novel aspect of the work, and might need to be better highlighted in the discussion.

**Response:** We highlighted the rural aspect of the sample in the introduction and discussion.

Line 386: typo

**Response:** Corrected.

Line 57: Typo

**Response:** Corrected.

**Reviewer #3:** The authors studied what successful aging means among 40 middle-aged Latinas who participated in focus groups conducted within the context of the CHAMACOS Study in the Salinas Valley of California in 2021-2022.

In the Introduction, the authors provided an adequate summary of the state of the field of successful aging research based on relevant published work, highlighted gaps in knowledge about Latino populations, and articulated the rationale for their work. In the Methods, the authors provided adequate description of participant recruitment, conduct of focus groups, and thematic content analysis of the qualitative data. As reported in the Results, the authors observed four salient themes in participants’ personal definitions of successful aging: 1) good health & accepting physical changes; 2) active lifestyle; 3) children’s wellbeing; and 4) independence. In the Discussion/Conclusion, the authors asserted that the evidence supports some unique views of successful aging for this Latina population relative to other populations, for example the importance of children doing well in life.

I do not have any major comments for improvement. The paper was well-organized and contained all necessary information to be a valuable contribution to the successful aging literature. I have only some minor comments below.

MINOR COMMENTS:
1. Abstract: In the Abstract Introduction paragraph, there appears to be an incomplete sentence with a question mark in the middle.

**Response:** We corrected this typo.

2. Results/Discussion/Conclusion: The statement of the four identified themes appeared at least three times – first paragraph of Results, first paragraph of Discussion, last paragraph of Discussion (Conclusion). As I reader, I felt this was redundant. Consider stating the list of four themes only once (in the Results), and then omit the list from the Discussion paragraphs but rather use the Discussion paragraphs to explore implications of the four themes.

**Response:** We edited the first paragraph of the discussion and conclusion to avoid repetition of the themes, and instead enhanced our discussion paragraphs to explore implications of the four themes.
