# Supplementary material for: Definitions of successful aging among middle-aged Latinas residing in a rural agricultural community
Source: PLoS One. 2023 Nov 30;18(11):e0294887. doi: 10.1371/journal.pone.0294887 (PMC10688629; doi:10.1371/journal.pone.0294887)
Supplement: Attachment — Submitted filename: Response to Reviewers_10.10.23.docx [file pone.0294887.s006.docx]

**Response to Reviewers**

Journal Requirements:

Please review your reference list to ensure that it is complete and correct. If you have cited papers that have been retracted, please include the rationale for doing so in the manuscript text, or remove these references and replace them with relevant current references. Any changes to the reference list should be mentioned in the rebuttal letter that accompanies your revised manuscript. If you need to cite a retracted article, indicate the article’s retracted status in the References list and also include a citation and full reference for the retraction notice.

**Response**

We updated the reference list to include relevant current references. We specifically updated reference #34. If you believe there are others that are retracted, we would appreciate it if you could help us identify them.

6. Review Comments to the Author

Please use the space provided to explain your answers to the questions above. You may also include additional comments for the author, including concerns about dual publication, research ethics, or publication ethics. (Please upload your review as an attachment if it exceeds 20,000 characters)

Reviewer #4: Please see attached file.

**Major comment**

The premise of the study of highlighting the role played by psycho-social-cultural factors involved in the perceptions of ‘successful aging’, and its findings as potential tools to promote and guide policies leading to achieve healthy aging, are valuable complements of the biological perspective on aging.

Although the present study raises an important point, for instance, the impact on the quality of life of the participating women exerted by their children wellbeing, it is also true that if something aggravating happens to a child it will be followed by an impact on mothers’ biology, e.g., immune system, making them more prone to disease and degradation of life quality. The lack of explicit recognition of the tight interconnections between individual/group perceptions of successful aging and the underlying biology of health and disease is what this paper is missing. This recognition strengthens rather than weakening the premise of the study while recognizing the complexity of the topic, i.e., ‘successful aging’, that this paper approaches. This is why this Reviewer notes that the approach utilized in this study is a ‘valuable complement’ of the biological perspective on aging.

In regard of this, a recent investigation on cognitive decline and dementia in the Multi-Ethnic Study of Atherosclerosis (MESA) comprising 1036 participants of Black, Chinese-American, Hispanic and White origin, of which 53% were women, showed that it is not race/ethnicity but cardiovascular risk factors such as age, smoking, hypertension, diabetes and socio-economic status, that determine the outcome on cognitive decline (Austin, T.R. et al., 2022. JAHA).

**Response**

We appreciate this comment and have explicitly addressed the connection between individual/group perceptions of wellbeing/successful aging and the underlying biology of health and disease in the discussion section. We have also highlighted existing research that highlights the connection between biopsychosocial factors and health. This recognition strengthens our paper and the complexity of the topic of successful aging.
